# Understanding the Use of the Term “Weaponized Autism” in An Alt-Right Social Media Platform

**DOI:** 10.1007/s10803-022-05701-0

**Published:** 2022-08-10

**Authors:** Christie Welch, Lili Senman, Rachel Loftin, Christian Picciolini, John Robison, Alexander Westphal, Barbara Perry, Jenny Nguyen, Patrick Jachyra, Suzanne Stevenson, Jai Aggarwal, Sachindri Wijekoon, Simon Baron-Cohen, Melanie Penner

**Affiliations:** 1 University of Toronto, 500 University Ave, M5G 1V7 Toronto, ON Canada; 2 Bloorview Research Institute, 150 Kilgour Rd, M4G 1R8 Toronto, ON Canada; 3 Department of Psychiatry and Behavioral Sciences, Feinberg School of Medicine, Northwestern University, 60611 Chicago, IL USA; 4 Chicago, IL USA; 5 William & Mary Williamsburg, 23185 Williamsburg, VA USA; 6 Child Study Center, Department of Psychiatry, Yale University, 06520 New Haven, CT USA; 7 Ontario Tech University, L1G 0C5 Oshawa, ON Canada; 8 The Palatine Centre, Durham University, Stockton Road, DH1 3LE Durham, UK; 9 University of Toronto, 6 King’s College Road, M5S 3G4 Toronto, ON Canada; 10 Department of Psychiatry, University of Cambridge Autism Research Centre, Douglas House 18b Trumpington Road, CB2 8AH Cambridge, UK

**Keywords:** Autism, Hate speech, Discourse analysis, Alt-right, Social media

## Abstract

**Background:**

The term “weaponized autism” is frequently used on extremist platforms. To better understand this, we conducted a discourse analysis of posts on Gab, an alt-right social media platform.

**Methods:**

We analyzed 711 posts spanning 2018–2019 and filtered for variations on the term “weaponized autism”.

**Results:**

This term is used mainly by non-autistic Gab users. It refers to exploitation of perceived talents and vulnerabilities of “Weaponized autists”, described as all-powerful masters-of-technology who are devoid of social skills.

**Conclusions:**

The term “weaponized autism” is simultaneously glorified and derogatory. For some autistic people, the partial acceptance offered within this community may be preferable to lack of acceptance offered in society, which speaks to improving societal acceptance as a prevention effort.

## Introduction

Online hate is a growing concern (Conway et al., 2019), which has led to increasing focus on understanding why people engage with this material. Of particular concern is the use of the term “weaponized autism” on social media platforms and forums associated with hate. The term “weaponized autism” is defined as “the focused application of nerdiness, computer tech savvy, and social awkwardness in the cyber pursuit of justice, payback, or even serving the public interest” (BarrySoetero, 2016). To date, our understanding of what is meant by “weaponized autism” has only been informed by a few media mentions and blogs (Borrell, 2020; DEO, 2017), with no systematic study. Understanding the meanings and implications of such terminology is important for three reasons: to identify potential vulnerabilities for autistic people on the Internet; to inform prevention and deterrence efforts for engagement with online hate; and to challenge stigma that comes from associating autism with violence.

Increasingly, people across the globe are using online spaces to connect with others and to form communities (Bateman et al., 2011). This is particularly true for autistic people, who often find that platforms designed for online connection and expression match their communication strengths (Gillespie-Lynch, 2014). Many autistic people report increased comfort, confidence, and ability to socially connect with others when interacting online compared to in-person interactions (Dekker, 2020; Gillespie-Lynch, 2014; Leadbitter et al., 2021; Shattuck et al., 2011). This contributes to a growing trend for autistic people to seek out online spaces for a sense of belonging to a community (Gillespie-Lynch, 2014; Leadbitter et al., 2021).

Concurrent with this trend, online hate speech is proliferating at a dramatic rate (Conway et al., 2019; Perry & Olsson, 2009; Perry & Scrivens, 2016). There is no universally agreed term for categorizing the online users whose discourse we have examined for this paper. Many of these online users self-identify as “alt-right”, and this term is often used in relevant literature (Donovan et al., 2018; Greene, 2019). However, we think that terms such as “fascist,” “xenophobic,” “neo-Nazi,” “white supremacist,” and “white nationalist” may sometimes also apply. For this paper, we attempt to define “hate” by drawing on the work of hate studies scholars Schweppe & Perry (2021), who acknowledge that a universally accepted definition of hate is not available and potentially not possible or desirable given the complexities and contextual factors that surround its understanding. “Hate” is an ambiguous label that may seem to connote emotion, and certainly it sometimes presents with extreme emotion; however, hate also manifests as rational and structured patterns of oppression (Schweppe & Perry, 2021). At its core, hate is identity-based and serves to reinforce the presumed marginality of some people based on elements of their identity such as race, colour, religion, disability, sex, gender, sexual orientation or national origin (Schweppe & Perry, 2021). It is important to note that terminology around hate-based and extremist groups is fluid, since group insiders strategically change terminology in efforts to maintain an image that is more palatable to society (Donovan et al., 2018; Kennedy et al., 2018). The language we use in this article is reflective of current temporal and North American contexts and will need to be tested for relevance in other contexts.

Exposure to online hate propaganda is common among Internet users, especially young people (Oksanen et al., 2014). Individual risk of exposure to this type of messaging rises with increased online activity, decreased attachment to family, and experiences of bullying both online and offline (Oksanen et al., 2014), all of which may be more common for autistic people (Cappadocia et al., 2012; Kuo, 2014; Sofronoff et al., 2011). This trend is expected to grow as hate groups increasingly recruit for their movements in online platforms (Perry & Olsson, 2009). The combination of these trends greatly amplifies the risk for certain autistic people to become involved with hate-based groups, which comes with additional risks of engaging in offline behaviours that are potentially harmful to themselves and others.

While not well studied or documented in academic literature (our search of available literature uncovered only one source exploring this - see Lovett (2019) for an exploration of alt-right recruitment tactics), journalistic reports have indicated that hate groups are working online to actively seek and engage autistic people for recruitment (Borrell, 2020; DEO, 2017; Schroeder 2019). Autism advocacy groups and community service organizations have begun to voice similar concerns and are calling for an organized response that is based on evidence and informed by autistic people (Autism Against Fascism, (no date); Braune 2020).

Based on these concerns, our research team embarked on a multiphase, multinational study examining factors that influence some autistic people to engage with online hate-based material. While immersed in data for the larger study, we noted frequent use of the term “weaponized autism”, a term we had also come across in blogs and social media. Media descriptions of “weaponized autism” note that this term is often used positively in online hate spaces, and generally connotes that loosely defined features of autism can be useful against one’s enemies (DEO, 2017). The objective of the present study was to undertake a discourse analysis examining the use of the term “weaponized autism” on Gab, an online site associated with hate speech. We aimed to develop a better understanding of how “weaponized autism” reflects, impacts, and produces perceptions of autism within this space, and to understand how this term may impact people who identify as autistic when they are engaged in this discourse.

## Methods

### Approach: discourse analysis

We chose an approach to discourse analysis described by Crowe (2005), as it is well suited to our positions as researchers and practitioners (in occupational therapy, medicine, health, psychology, and sociology) and to the type of knowledge we wish to generate (to inform practice). Discourse analysis allows us to go beyond the surface meaning of the text and explore the symbolic or latent meaning of the term “weaponized autism” and explore where this term comes from, how it relates to social structures in “the real world” (Lupton, 1992) and how it impacts the experience of autistic people in online hate speech forums. Discourse analysis is useful to examine how acts of normalization and marginalization are performed through discussion (Korobov, 2020). Examination of digital discourse can facilitate an understanding of how discussion is used to enact identities, activities, and ideologies within a digital community, as part of a larger society (Gee, 2004).

### Research paradigm

The methods described here were undertaken within a larger study that adopts an epistemological stance of constructivism. This paradigm acknowledges that we are not objective observers separate from research (Annells, 1996); rather, we are part of the research, beginning with the formulation of the research question (Annells, 1996; Appleton & King, 2002). Our ontological position is one of relativism, which considers reality to be dynamic and context specific (Howell, 2012). Our decision to apply a discourse analysis recognizes that discursive texts are generated within a sociopolitical context and that our interpretations are subjective (Lupton, 1992). While it does not generate a “universal truth”, this approach can be used to inform practices for clinicians, policy makers, law enforcement and others who wish to support autistic people at risk for engagement with online hate-based materials.

### Researcher values

As a team, we share the following values: (1) that devaluing human beings based on race, colour, religion, disability, sex, gender, sexual orientation or national origin is harmful, abhorrent, and reflective of false logic; (2) that autism is a neurological variation that comes with many appreciable strengths as well as highly individualized challenges, dependent on disabling factors in the environment; and (3) that autistic people hold valuable insights to their own circumstances as well as the world around them, and many are interested and able to make important contributions to autism research. Our backgrounds, which include academic and clinical experience in occupational therapy, medicine, psychiatry, psychology, criminology, and sociology, as well as lived experiences of autism and extremist group involvement and radicalization/deradicalization practices, influence how we interpret the data.

### Description of data source: Gab

Gab is a social media platform which launched publicly in 2016 (Donovan et al., 2018). It proclaims itself to be “a social network that champions free speech, individual liberty and the free flow of information online” (Gab.com, 2021). It has been widely criticized by hate watch groups, journalists, and researchers as being a harbor for extremist and hate-based discourse (Donovan et al., 2018; Jasser et al., 2021). Jasser and colleagues (Jasser et al., 2021) conducted an analysis of Gab posts and found a high frequency of hate speech among users posting public messages, most commonly communicating anti-left, anti-Semitic, misogynistic, racist, and xenophobic sentiments. Gab is described as a small social media platform that combines features and functions of Facebook, Twitter, and Reddit, but is distinct from these mainstream platforms in that it offers almost no content moderation (Donovan et al., 2018; Jasser et al., 2021). This has made Gab a haven for social media users who have been banned from mainstream platforms for contravention of terms of service moderation.

### Data generation: data corpus

The original data corpus was generated by the Southern Poverty Law Center (SPLC) and shared with our research team. SPLC used a method called ‘scraping’ (Hookway, 2008), in which all public posts on discussion boards are captured and added to an offline dataset. Posts included date from November 2018 to March 2019 and were searched for a list of terms relevant to autism. The list of terms was compiled based on the collective expertise of our research team. All contributed terms were included in the final list: autism, ASD, autistic, PDD, Asperger’s, Aspie, ‘on the spectrum’, ‘spectrumy’, ‘sperg’, ‘sperging out’, ‘autist’, and ‘weaponized autism’.

### Data generation: subset of data used for this analysis

The subset of data used for this analysis was created by filtering the data corpus for posts that contain the term “weaponized autism” or variations of the term. To be included, the post had to contain two key words: “weapon*” and “autis*” (the * indicates that different characters can follow after the word). For example, “weaponized autist” and “weaponize autism” would both be included in the search. Posts where additional words in between, preceding or following “weapon*” and “autis*” were also included in the search (e.g., “Been weaponizing the shit out of my autism on all the new drops, we’ve got more tactical strikes coming Monday …”). The search also included posts where “autis*” came before “weapon*”. For example, phrases like “Autism can be a powerful weapon if harnessed for the forces of good." and “We cherish our autism for we have weaponized it and made of it a terrifying sword with which to harrow our enemies." — were also included in the search. This search generated a dataset totalling 711 posts.

### Data analysis

Informed by Crowe’s approach (2005), this discourse analysis used a multidisciplinary, multi-perspective lens, drawing on the varied clinical and academic expertise, as well as lived experiences of our research team (which includes lived experience of engaging in hate group activity and lived experience of autism). The first author (CW) was deeply immersed in the data and connected regularly throughout the analysis with the senior author (MP) to discuss significant points in the analysis. At midway and near the end of the analysis, the first author presented a summary of observations and progress on the data analysis to the full research team. The team then contributed to the analysis, providing additional insights, and helping to contextualize findings. This allowed us to leverage the varied kinds of knowledge of team members to confirm, validate, challenge, complicate, extend, and refine the analysis. Table 1 outlines important steps in the process as described by Crowe and related questions we used to interrogate the data and guide our analysis.

**Table 1:** Key questions throughout the analytic process

Step in Analytic Process (Crowe, 2005)	Key Questions
Select the text	What type of “speech act” is being analyzed? What is the significance of this? What is the context in which these Gab posts are produced?
Identify the explicit purpose of the text	What is the stated purpose of these Gab posts? What other purposes (larger or alternate or hidden) might motivate them? What do the users of this term gain or avoid?
Examine the processes used for claiming authority	How do Gab posters claim authority? Who has “power” in this space? How do users gain and exert power? What kind of knowledge or status is privileged in alt-right online spaces?
Explore connections to other discourses	How has this definition of autism been influenced by media, social media, and academic / medical literature? What values underpin the way Gab posters have defined / labeled autistic people?
Critique the construction of major concepts	What is missing from their definition of weaponized autism? What are they not saying? What part of “real life” is not being represented?
Name and categorize	What values and assumptions held by Gab posters are underlying the term “weaponized autism” and how it is used?
Examine the construction of subject positions, construction of reality and social relations	What does the use of this term “weaponized autism” say about attitudes and assumptions toward autism in this space? Where do these ideas come from? What are the underlying assumptions? How have these assumptions been promoted / propagated in society? How might the “echo chamber” effect (lack of counter argument) of this site shape a person?
Identify implications for practice	How might the use of the term “weaponized autism” impact autistic people within this space? What might be the impact as this term emerges into the mainstream / “real world”, particularly for autistic people and those who care about autistic people?

### Steps to enhance rigour

We took several steps to enhance the rigour of this study. As suggested by Greckhamer and Cilesiz (Greckhamer & Cilesiz, 2014), we approached the work in a highly planned and systematic way, employing the phased approach described by Crowe (2005) and explicitly identifying *a priori* questions we would use to interrogate the data. We also documented the process using a research diary, analytic memoing and transcripts from team meetings. As recommended by Crowe (2005), we clearly state the epistemological and ontological basis for this study and how it fits with discourse analysis, acknowledging discourse analysis as an interpretive process. In our reporting, we strive for transparency by providing a detailed account of our process and analytic procedures, as well as sufficient samples from the data to support our claims (Greckhamer & Cilesiz, 2014).

### Ethical considerations

While the use of extant texts that have been scraped from online discourse can be seen as “non-invasive”, there remain important ethical considerations for this study. Eynon, Fry and Schroeder (Eynon et al., 2017) propose three concepts at the core of research ethics: confidentiality, anonymity, and informed consent. Maintaining confidentiality and anonymity was a straightforward process, as we did not gain access to the “real” identities of Gab users. Additionally, we do not share usernames when reporting on this work. While informed consent is important in human subject research, it is well accepted that certain kinds of research can ethically proceed without informed consent, including observational research in public places and analysis of texts that are “in the public domain” (Willis, 2019).

This raises the question of what is considered “public domain” online. According to Willis (Willis, 2019), different online forums have different levels of “publicness” and this level of “publicness” or privacy perceived by the online user can be measured by two factors: (1) how technically accessible the platform is and, (2) how the forum users intend the text to be used. Applying these measures to Gab posts, we see that the technical accessibility is very high. In order to sign up for Gab, prospective users only need to provide a username and an email address. Additionally, anyone visiting the site can view content on Gab without becoming a registered user, using a search function on the homepage. This would suggest a high level of perceived “publicness” and low level of privacy according to technical access. With respect to how Gab posters intend the information to be used, they are directing their content at fellow Gab users, not at researchers, policy makers or the general public. However, within our data corpus, Gab posters express an awareness within their posts that people outside of what they call the “Gabfam” sometimes read Gab content and that privacy cannot be assumed when posting on Gab. Gab’s “terms of use” state that registered users must be at least 18 years of age; however, we cannot confirm that users abide by this rule.

Our team has also been concerned with the ethics surrounding reporting and dissemination of our findings from this study and from our larger study. We are aware of the potential to cause harm in the greater autistic community by highlighting an association between autism and engagement with online hate material, when our best information suggests that autistic people are much more likely to be targets of online aggression than perpetrators (Ashburner et al., 2019; Campbell et al., 2017). We have attempted to mitigate this concern with careful use of language in our reporting, with special attention to suggestions made by our autistic coauthor (JR).

This study received approval from the Holland Bloorview Kids Rehabilitation Hospital Research Ethics Board.

## Results and discussion

This analysis combines targeted study of a specific discourse with a broader study of its context, as is customary in discourse analysis (Crowe, 2005). We therefore present the findings and the discussion together in this section.

### A complicated analysis

 Our analysis was complicated by obfuscating language used within Gab, which is among several known communication tactics in alt-right spaces (May & Feldman, 2018). Gab, like many online spaces dedicated to alt-right or white nationalist interests, deliberately use humour, sarcasm, irony, and “playful” imagery (including memes) to obscure their intentions to spread hate (May & Feldman, 2018). These so-called “LULZ” (an adaptation of laugh out loud, or LOL) tactics are employed to rebrand and obfuscate fascist ideology and allows users a level of deniability when called out for their hate. These tactics work to make their offensive material more palatable to the mainstream, enhance their recruitment of young people and people who would be repelled by overtly violent or racist material, facilitate identification of insiders (those who know the vernacular) and outsiders (those who do not), and ultimately allow groups to “hide in plain sight” (May & Feldman, 2018). These tactics also pose a challenge to discourse analysis. Humour, sarcasm, and layers of irony are designed to leave many statements “open to interpretation”. Because of these tactics, extended immersion in the data was required to develop confidence in detecting the intended message of a post.

The questions with which we interrogated the data can be collapsed into three meta-questions: (1) What does this term mean? (2) Where does it come from? and (3) What does it enact? We break down our analytic findings along these same lines: (1) the meanings of the term in this context; (2) the roots of the term and the realities it reflects; and (3) the ways in which the term impacts people and how it shapes reality (within the “#Gabfam” and beyond).

### Understanding the term “weaponized autism” and its meaning in Gab context

When conducting discourse analysis, it is critical to consider “who is talking” and “who is not” (Crowe, 2005). Within our dataset, “weaponized autists” are frequently discussed but rarely part of the discussion. Of the 711 posts included in this analysis, only 19 are written by people who claim to be autistic or possess “weaponized autism.” These 19 posts were generated by nine different users. Most discussion of weaponized autism refers to users among “the chans” (online unmoderated, anonymous forum sites such as 4chan and 8chan) or referencing unidentified people behind certain stunts or feats that the posters have learned of through media. Weaponized autists are frequently *spoken of* and occasionally *spoken to*, usually with some sort of call to action.

*I need the weaponized Autism of /pol/! /pol/ got a president elected. Now we need a party in congress that will support him! #MAGAParty* *NOTE /pol/ is a reference to “politically incorrect”, a discussion board on 4chan known for hate speech (Tuters, 2018)

Well thought out! Now, get the weaponized autists at 4Chan in on this!

The Gab definition of weaponized autism is rooted in an esoteric understanding of autism itself. When Gab posters speak of autistic people, they are not necessarily referring to someone who has been diagnosed as autistic or meets the diagnostic criteria if assessed. Rather, they are applying a stereotype of autism, which is largely of their own making, but has roots in medical and media representations of autism. Their autism archetype is a person with hyper-focus, social awkwardness, ‘savant’ or very narrow intelligence, a specific or specialized talent, and is usually single (probably a virgin) and “NEET” (not in education, employment, or training).

*If you click on the link, the title of the photo is “Behold the power of weaponized autism.“ A lot of these guys are geeky, tech savvy, and a bit autistic(if not a lot).*

*In a nutshell many of them are basically super-geeks with some pretty awesome technical skillz. They tend to be politically conservative. You don’t want to get on their bad side :O*

Each of these posts collapse autism with both social ineptitude and technical competencies. Interestingly, the reference to the risk of being on their “bad side” enables the weaponization: it is this implied potential for retaliation along with technical skills that makes autistic people useful to the movement.

Building off the Gab autism archetype, weaponized autism is the harnessing of hyper-focus and talents of “autistic” people. This “weapon” can be used to advance the interests of the alt-right, including harming their enemies, opponents, or people they see as undesirable.

* weaponized autism. The focused application of nerdiness, computer tech savvy and social awkwardness in the cyber pursuit of justice, Payback or even serving the public interest*

*WEAPONIZED AUTISM!!! :D Now we know the evolutionary purpose for autism: highly focused warriors who relentlessly pursue the enemy.*

*It’s called weaponized autism, it’s the waxing and waning of shit posting intermixed with real life, math, philosophy, and smart LARPing.*

*Autists can be the most fanatical people you can find. We need “Waffen-Autismus” aka Weaponized Autism. Give those people a cause and something their skills are useful for and watch things get awesome.* *NOTE “Waffen” is the German word for “weapons” and is also employed in the name of “Atomwaffen Division,” a defunct neo-Nazi network.

For these posters, autism is a readily exploitable resource that can be channelled in pursuit of their agenda, and especially as a means for lashing out at those who are considered “the enemy.” Indeed, Gab posters within our data discuss how autistic people can be deployed to advance the interests of the alt-right. The comments reveal a sometimes implicit, other times explicit assumption that autistic people are ripe for manipulation.

*The greatest weapon on Earth is the weaponization of the depressed and emotionally vulnerable. Is Weaponized Autism not a piratical suicide delivery device? Can Social Media be used to find and manipulate useful idiots? Sadly yes*

*Back in the day (2016) we on the very hard right, the white nationalist alt-right, used these people as a force multiplier because at the time we desperately needed their weaponized autism to elect Trump.*

What makes weaponized autism so appealing to Gab posters is that it is seen as an all-powerful, omniscient force with limitless reach. It is highly prized as a sophisticated and advanced tool in the alt-right arsenal and perceived as an “unstoppable” means by which to secure the salvation of white, western culture.

*So few really appreciate the pure raw POWER of weaponized Autism! It’s reach is limitless, it’s power isinfinite!*

*My god, if #CrookedHillary really wants to control the people and stop #Trump, she need to stop worrying about guns and ban weaponized autism ASAP. Their autism know no bounds. #MAGA*

*This is amazing! No other superpower in the world can stand against weaponized autism!*

*We are only beginning to scratch the surface of weaponized autism. Even if we have harnessed a fraction of its power, we will be nigh on unstoppable.*

*We’ve weaponized autism and it’s both beautiful and terrifying to behold.*

Weaponized autism is seen as particularly valuable to the alt-right because it operates in what the posters see as their primary battlefield (the Internet) and is appropriate for the type of conflict they prize (meme warfare). Autists are thought to excel in the development and circulation of memes that signal insider/outsider status, and that communicate the aims of the alt-right.

*Let us praise all the fine meme war soldiers on the Right side of history! Your weaponized autism is appreciated and will not be forgotten*

*Normie Oppression will never end!!! WEAPONIZE YOUR AUTISM This is the MOTHER OF ALL MEME BATTLES.*

These posts suggest that “meme battles” are central to their tactics, and the greatest warriors are thus autistic people. Weaponized autism, then, is also considered to be something uniquely owned by the far right. It is claimed as something that has been and will be helpful in advancing their agenda, which is sometimes overtly linked to promoting favoured political figures and defeating their political enemies.

*I love this. Obama may have weaponized stupidity, but Trump supporters at r/The_Donald have weaponized autism.* *NOTE r/The_Donald/ is a reference to a subreddit that has since been banned by Reddit for not conforming to their standards (Lima, 2019).

* I amconvinced our only plan for victory is convincingly smearing every Dem in 2018 as some type of Sex Offender, GOP obviously won’t do that so we need weaponized autists to spread #pizzagate times 10, but more convincing.* *NOTE “pizzagate” is a reference to a conspiracy theory that claimed Hillary Clinton ran a child-sex ring in a pizzeria.

While weaponized autism is often discussed in terms of direct political advancement, it is also discussed as a tool for general mischief making and “entertainment.” “Shitposting” and other forms of online pranking that capitalize on diligent detail-oriented research (such as identifying people of interest) are among the LULZ strategies and other tactics discussed earlier, which are employed to simultaneously demonstrate the group’s power and distract from their most nefarious work and intentions.

*BOOM!!!!! Shitposting has now reached fully weaponized autistic levels!!!!*

*Saturday is April Fool’s Day and I suggest we make use of it. I want every Gabber, Alt-Right and conspiracy Theorist dropping round after round of weaponized autism on the enemy via social media.*

*Later you can claim it was just a joke…#MAGA #GABFAM*

To be a weaponized autist is to be valued. Weaponized autists are held up as heroes within the Gab posts. Many posters express admiration, appreciation, and affection for these “soldiers” for their cause.

*Weaponized autism is a wonder to behold to us regular people. You guys are thermonuclear! Thank you for never giving up on anything!*

*Who’d have thought the heros of the day would be the weaponized autists of 4chan? Not only are they successfully identifying cowardly masked #Antifa anarchists, but they have analyzed their mob tactics in detail. These guys deserve some love!*

*Apparently it’s World Autism Awareness Day - the AltRight is ahead of the SJWs [Social Justice Warriors] when it comes to this. The AltRight respects and cultivates the awesome power of Weaponised Autism, this gives meaning and direction to many autist’s lives. #Gabfam #Maga #Trump*

*Calling other [people] “weaponized autists” is a high honor bestowed on those who fight for good here :)*

Paradoxically, to be a weaponized autist is to be simultaneously devalued. Alongside the expressions of admiration and affection are incongruous sentiments of degradation. While useful and talented, weaponized autists are seen as “strange” and incapable of many “normal” tasks such as maintaining hygiene, relationships, and employment. They are presumed to spend time engaged in “useless” activities (such as online gaming) and to occasionally engage in “unusual” behaviour, such as making screeching “Reee” sounds.

*Our top Kek priests are in development of a new kind of weapon... Called the Reeeening Rifle. Shoots dank memes with semi-auto, and concentrated weaponized autism with fully-auto.* *NOTE “Kek” is an insider vernacular term with several meanings, in this case referring to a fictional culture / heritage and religion shared by alt right online community (Tuters, 2018)

* Never underestimate weaponized autism and the boredom of a couple of unwashed NEETS.*

The ambivalence directed toward weaponized autists is reflective of their alt-right values. The simultaneous valuing and devaluing of weaponized autists is elucidated when their autism archetype is mapped onto values held by the group. The alt-right are known to value whiteness, stereotypic maleness, cisgender, heteronormativity, and ‘rational’ intellect (or pseudo-intellect) (Bogerts & Fielitz, 2019; Forscher & Kteily, 2020; Nilan, 2021) and these characteristics match their stereotype of autistic people. Alt-right ideology also values power, strength, capitalistic prominence, social dominance, sexual access to women, and being head of a household (Bogerts & Fielitz, 2019; Forscher & Kteily, 2020; Nilan, 2021), all of which are lacking from their weaponized autist archetype.

### Roots of the term weaponized autism and the realities it reflects

The Gab archetype of an autist did not emerge in a vacuum. Rather, it is rooted in some portrayals of autism in media. As mentioned earlier, a weaponized autist, as discussed within Gab, is not necessarily a person with a formal diagnosis (though it can be). The image portrayed within Gab aligns closely with the cultural figure of “nerds’’ depicted in popular culture; specifically, a person who is male, who fails to fit hegemonic standards of masculinity, a computer user, is technologically brilliant, but is socially inept (Kendall, 1999). In addition to the “nerd” stereotype, the media has offered many portrayals of autism. Journalistic portrayals of autism have tended to offer a negative or tragic view of autism and to portray autistic people as asocial, or even antisocial (Huws & Jones, 2011). Early entertainment-media portrayals of autistic people have been criticized for depicting people who were either subhuman or superhuman with nothing in between (Maich, 2014). Today, autistic characters are featured in a growing number of movies and television series (Nordahl-Hansen, 2017). Despite the growing popularity of autistic characters, autistic people are generally left out of both the production and the casting of these characters, and the characters are criticized as being archetypal rather than authentic (Nordahl-Hansen, 2017) with said archetype often matching that which is portrayed in Gab.

It is also the case that the Gab archetype of an autistic person is rooted in some portrayals of autism in medicine. With a growing portion of autism research being informed by a neurodiversity framework, we are developing an improved understanding of the heterogeneity of autistic people, beyond simply thinking of “levels of severity” (Welch et al., 2020). This includes explorations of autistic experience that challenge older, dominant conceptualizations of autism. However, early framing of autism maps well on to the reductionist Gab autism stereotype. Especially fitting is the former diagnostic category of Asperger Syndrome, which was named after Hans Asperger, who reportedly referred to his young patients as “little professors”, comparing their knowledge (relevant to their area of interest) to that of erudite professors, but also describing them as socially unskilled, unaware, and uninterested (Osborne, 2002).

The term “weaponized autism” might also be understood as an extension of the broader use of militarized speech in this forum. The term aligns with the general tendency by users on Gab to use militarized language on this site. This is reflective of the alt-right value of authoritarianism (Phillips & Yi, 2018), as well as a collective sense of grievance and an “us against the world” mentality frequently intermingled with the idea of being at war with much of the world (Forscher & Kteily, 2020). Presumably, posters use militaristic speech to evoke a sense of power / authority and legitimacy as well as being precise and well organized, all things that are valued in this space (Nilan, 2021; Phillips & Yi, 2018).

*We cherish our autism for we have weaponized it and made of it a terrifying sword with which to harrow our enemies*

*The side effects of weaponized autism were still not fully understood in 2017, much like the side effects of nuclear weapons in 1945*

The use of the term “weaponized autism,” then, is not out of line with standard discursive practices of the alt-right, indeed, to “weaponize” something is itself a common alt-right strategy. That is to say, they capitalize on opportunities to advance their agenda by manipulating people, sentiments, media, technology and events for their own purposes (Ganesh, 2020; Munn, 2019; Picciolini, 2020). The alt-right have been known to “weaponize” online memes (Ebner, 2019), cartoons (Bogerts & Fielitz, 2019), irony and idioms (Albrecht, 2019), and use technology such as YouTube recommendations (Munn, 2019) to advance their agenda.

### How use of the term “weaponized autism” shapes reality (within the “#Gabfam” and beyond)

Within the #Gabfam, the simultaneous valuing and devaluing of weaponized autists are internalized and reflected in the posts of Gab users who identify as weaponized autists. Some of the posts from users who self-identify as weaponized autists reflect a sense of pride, accomplishment, and positive identity. It is a badge of honour. These posts mirror the high regard for weaponized autists that is sometimes conveyed by other Gab users. Other posts from self-identified weaponized autists reflect an internalization of the negative stereotypes attached to this label.

*you don’t understand I AMweaponized autism. I AMthe memetic autistic nonsense. it’s thanks to autists like me that Trump is your President (if you’re American), it’s autists like me that keep balance within the universe. you’re welcome*

*Ah dude its the nature of being a weaponized autist_x000D_ It destroys sanity and replaces with raw memes*

*actually that makes sense. thanks m8 that’s why I don’t have anyone, no one deserve to be paired up with the pure weaponized autism that I am :D I’m okay with this, I’m a living weapon :D*

Unfortunately, the #Gabfam’s level of acceptance for some autistic people may be better than that of general society. A critical contextual factor to consider is that many autistic people experience a high level of rejection in general society (Acker et al., 2018), such that the partial or intermittent acceptance offered to autistic people in the Gab space will, to some people, be preferable to the general non-acceptance experienced in larger society. Within the “#Gabfam”, autistic people often have insider status and an important role to play. This role is seen as critical to the cause and this cause is central to this community. While the term is partly insulting and derogatory, it is also partly glorified, and it speaks of a person as talented, smart, and useful. Additionally, many autistic people encounter challenges developing close, reciprocal relationships and commonly experience maltreatment within relationships (Pearson et al., 2020). This can make the maltreatment encountered on Gab seem “normal”. The quotes below highlight pervasive experiences of rejection in greater society as well as experiences of acceptance and a role to play within Gab.

*I just like it when we do agree - it means I am not a complete lunatic. I like it when I agree with anyone. Keeps me sane knowing Im actually not alone. Being a weaponized autist has drawbacks, and isolation and depression are 2*

*Lol Ive been called worse. Im 99% pure weaponized autism. If it werent for the 1% not weaponized I would have imploded into a black hole by now. I know I will be alone. I do not expect ANYTHING in return. My fate is sealed. I am at peace with this role.*

*Im ugly, and none of those other things. My extremely dense weaponized autism is as bad as radioactive waste for womens’ health. I stay single for their sake. They dont wanna deal with this level of autism*

Ultimately, Gab creates an “echo chamber” that shapes users, including autistic users. Gab is specifically designed for people to express extreme views in a forum where it will not be challenged (Jasser et al., 2021). Additionally, Gab discourse has been found to be driven by “super participants” who work to ensure dissenting / divergent views are discouraged (Zhou et al., 2019). “Intellectual” rationalizations for alt-right ideals are often presented in these forums, offered with a logic that can be very convincing, especially when there are no counter balancing arguments offered (Darmstadt et al., 2019). Posters whose content expresses white nationalist sentiments are often encouraged and praised; posters who express dissent are harshly criticized or worse, ignored (Jasser et al., 2021). Patterns of acceptance and rejection within discourse always have the power to shape people (Crowe, 2005). The acutely explicit and strong signals of acceptance and rejection seen on Gab are likely to be especially powerful.

### Implications

Our paper raises many considerations for autistic people, family members, researchers, clinicians, and policy makers. The term “weaponized autism”, and the communication tactics in which it is grounded, pose risks to autistic people, particularly autistic youth. There are many components to this risk. The Identity, Community and Purpose Model of Radical Socialization (ICP) suggests that attraction to hate based material is driven by unmet social needs rather than by direct appeal of the ideologies themselves (Picciolini, 2020). As the title of this model suggests, the primary unmet social needs are a sense of identity, a sense of community, and a sense of purpose. These three social needs are easily mapped on to the construct of weaponized autism as presented in the Gab posts. By becoming a weaponized autist, an autistic person is bestowed a clear identity, becomes an insider to this community and has a vital and highly specialized role to play. The partial inclusion offered by the Gab community is better than the exclusion often enacted by larger society. Larger society poses many barriers for autistic people to attain a sense of identity, community, and purpose (Acker et al., 2018; Cappadocia et al., 2012; Pearson et al., 2022; Sofronoff et al., 2011). The marginalization experienced by autistic people can set them up to be responsive to the allure of the sense of identity, community and purpose offered to weaponized autists within Gab.

The layers of irony, as well as the humour and sarcasm used within these spaces (May & Feldman, 2018) increase the risk of attracting online users who are merely curious or who are attracted to the humour and “shenanigans” but who are not truly interested in the ideological underpinnings of alt-right forums. These layers of irony and humour can also make it difficult for family members and clinicians to know how entrenched a person is in the ideology, since the plausible deniability lent by LULZ tactics extend to individual users and people who consume the material.

It is not uncommon for “insider” vernacular terms, ideas, or memes to leak from niche platforms into the mainstream (Literat & van den Berg, 2019). Currently, the term weaponized autism can be found in public forums such as Reddit, Twitter, and other online blog posts. T-shirts, stickers, and buttons emblazoned with the term are available for purchase on Amazon and Etsy. Growing popularity of the term has the potential to cause harm to the larger autism community by suggesting an association between autism and violence, while simultaneously suggesting that autistic people are like “programmable robots”, or malleable and ripe for manipulation.

This remains an understudied area. Much thoughtful research is needed in order to better understand the engagement and recruitment tactics of alt-right and other hate-based groups directed at autistic people. Additionally, more work is needed to identify risk and protective factors to prevent and respond to autistic peoples’ engagement in online hate-based materials. This is especially crucial for autistic young people.

### Limitations

There are important limitations to this study and the way its findings can be applied. The posts scraped for this analysis were generated within or before March 2019. Given the way in which language is rapidly manipulated and frequently changed within alt-right discourse (Donovan et al., 2018; Kennedy et al., 2018), the use of the term “weaponized autism” can be expected to evolve. In fact, it is possible that this has already happened by the time of publication of this manuscript. It must also be considered that each online platform / forum has its own unique culture and vernacular, and that the nuanced interpretations offered here are specific to Gab and the term “weaponized autism” may have somewhat different nuance within discourse generated on other forums, including alt-right spaces. The online posts scraped for this analysis were limited to those containing actual use of the term “weaponized autism” (or a variant of the term) and did not contain full threads of conversation for analysis. This limits some of the contextual information for these posts and may have impacted the interpretations we have offered.

## Conclusions

This analysis enhances our understanding of the term “weaponized autism” as used within Gab. We see the term as reflective of a reductive autism archetype of their (Gab users) own making and used to refer to people who may or may not actually be autistic. The term “weaponized autism”, as used within this space, has both positive and negative connotations, but could be seen by some autistic people as net-positive, especially if they experience a high level of rejection in larger society and experience at least partial acceptance on Gab. This net-positive could pose added risk of engagement / recruitment for certain autistic persons looking for a sense of identity, community and belonging. Use of the term “weaponized autism” is in keeping with recruitment and communication tactics typical of alt-right groups. Ongoing use of the term “weaponized autism” poses risk of harm to autistic people within spaces such as Gab and in society. This issue is complex and highly nuanced. We strongly caution against reductive or sensationalistic dissemination of these findings, as this will exacerbate rather than mitigate the harms of the term “weaponized autism” for autistic people.

## Data Availability

Due to the highly sensitive nature of this data set, access to data will be considered upon request.
